# Cyanobacteria Respond to Low Levels of Ethylene

**DOI:** 10.3389/fpls.2019.00950

**Published:** 2019-07-30

**Authors:** Cidney J. Allen, Randy F. Lacey, Alixandri B. Binder Bickford, C. Payton Beshears, Christopher J. Gilmartin, Brad M. Binder

**Affiliations:** ^1^Department of Biochemistry & Cellular and Molecular Biology, The University of Tennessee, Knoxville, Knoxville, TN, United States; ^2^West High School, Knoxville, TN, United States; ^3^Farragut High School, Knoxville, TN, United States

**Keywords:** cyanobacteria, ethylene receptor, ethylene binding, Synechocystis, Geitlerinema, phototaxis, biofilm

## Abstract

Ethylene is a gas that has long been known to act as a plant hormone. We recently showed that a cyanobacterium, *Synechocystis* sp. PCC 6803 (Synechocystis) contains an ethylene receptor (SynEtr1) that regulates cell surface and extracellular components leading to altered phototaxis and biofilm formation. To determine whether other cyanobacteria respond to ethylene, we examined the effects of exogenous ethylene on phototaxis of the filamentous cyanobacterium, *Geitlerinema* sp. PCC 7105 (Geitlerinema). A search of the Geitlerinema genome suggests that two genes encode proteins that contain an ethylene binding domain and Geitlerinema cells have previously been shown to bind ethylene. We call these genes *GeiEtr1* and *GeiEtr2* and show that in air both are expressed. Treatment with ethylene decreases the abundance of *GeiEtr1* transcripts. Treatment of Geitlerinema with 1000 nL L^–1^ ethylene affected the phototaxis response to white light as well as monochromatic red light, but not blue or green light. This is in contrast to Synechocystis where we previously found ethylene affected phototaxis to all three colors. We also demonstrate that application of ethylene down to 8 nL L^–1^ stimulates phototaxis of both cyanobacteria as well as biofilm formation of Synechocystis. We formerly demonstrated that the transcript levels of *slr1214* and *CsiR1* in Synechocystis are reduced by treatment with 1000 nL L^–1^ ethylene. Here we show that application of ethylene down to 1 nL L^–1^ causes a reduction in *CsiR1* abundance. This is below the threshold for most ethylene responses documented in plants. By contrast, *slr1214* is unaffected by this low level of ethylene and only shows a reduction in transcript abundance at the highest ethylene level used. Thus, cyanobacteria are very sensitive to ethylene. However, the dose-binding characteristics of ethylene binding to Geitlerinema and Synechocystis cells as well as to the ethylene binding domain of SynEtr1 heterologously expressed in yeast, are similar to what has been reported for plants and exogenously expressed ethylene receptors from plants. These data are consistent with a model where signal amplification is occurring at the level of the receptors.

## Introduction

Ethylene is an important plant hormone that affects plant growth, development, and responses to many stresses (Mattoo and Suttle, 1991; Abeles et al., 1992). Ethylene receptors in plants have been studied for many decades and much is known about how they bind ethylene and signal to down-stream signaling proteins (Merchante et al., 2013; Lacey and Binder, 2014; Bakshi et al., 2015). These receptors, as well as several other plant hormone receptors, have homology to bacterial two-component receptors that signal via a histidine autophosphorylation followed by phosphotransfer to downstream targets (Chang et al., 1993; Schaller et al., 2011; Kabbara et al., 2018). Several research groups have proposed that plants acquired these two-component-like receptors from the cyanobacterium that gave rise to chloroplasts where the free-living cyanobacterium became an endosymbiont and most of the bacterial genome was acquired by the host cell (Kehoe and Grossman, 1996; Martin et al., 2002; Mount and Chang, 2002; Timmis et al., 2004; Schaller et al., 2011). In support of this, some cyanobacteria contain predicted ethylene binding proteins and several cyanobacteria species have been documented to bind ethylene (Rodriguez et al., 1999; Mount and Chang, 2002; Wang et al., 2006).

Many microorganisms respond to ethylene (Abeles et al., 1992; Bakshi and Binder, 2018). However, it was only recently that a non-plant species, *Synechocystis* sp. PCC 6803 (hereafter referred to as Synechocystis), was documented to contain a functional ethylene receptor (Lacey and Binder, 2016). In this unicellular cyanobacterium, the receptor is encoded by the *slr1212* gene locus and has been variously referred to as *Ethylene response 1* (*SynEtr1*) (Kaneko et al., 1996; Ulijasz et al., 2009; Lacey and Binder, 2016), *His-kinase44* (*Hik44*) (Los et al., 2008), *Positive phototaxisA* (Narikawa et al., 2011) (*PixA*), and *UV intensity response Sensor* (*UirS*) (Song et al., 2011; Ramakrishnan and Tabor, 2016). It is the only gene in the Synechocystis genome predicted to encode a protein with an ethylene binding domain. The protein contains a functional ethylene binding domain at the N-terminus followed by a phytochrome-like domain known as a cyanobacteriochrome, and a his-kinase domain at the C-terminus (Yoshihara et al., 2004; Ikeuchi and Ishizuka, 2008; Ulijasz et al., 2009; Narikawa et al., 2011; Song et al., 2011; Lacey and Binder, 2016; Ramakrishnan and Tabor, 2016). Thus, this is a receptor for both light and ethylene. Various studies have also delineated components of the signaling pathway downstream of the receptor (Narikawa et al., 2011; Song et al., 2011; Lacey and Binder, 2016; Ramakrishnan and Tabor, 2016). From these studies a model for light signal transduction from SynEtr1 has developed (Ramakrishnan and Tabor, 2016) where UV-A light stimulates histidine autophosphorylation followed by phosphotransfer to a conserved aspartate on the slr1213 response regulator protein. The phosphorylated slr1213 enhances the transcription of a small, non-coding RNA called *CsiR1* and *slr1214* which encodes a second response regulator protein. In contrast to UV-A light, application of 1000 nL L^–1^ ethylene reduces the transcript abundance of *CsiR1* and *slr1214* (Lacey et al., 2018), but it is not known whether or not this occurs via regulation of SynEtr1 histidine kinase activity. Ethylene also causes changes in the cell surface of Synechocystis cells leading to enhanced biofilm formation, more directed motility of single cells in response to directional light, and faster phototaxis when the cells aggregate (Lacey and Binder, 2016; Kuchmina et al., 2017; Lacey et al., 2018).

Many additional putative ethylene receptors have been identified in a wide array of bacteria and several non-plant eukaryotes (Mount and Chang, 2002; Wang et al., 2006; Lacey and Binder, 2016; Hérivaux et al., 2017; Kabbara et al., 2018), but it remains to be determined whether or not other cyanobacteria respond to ethylene. With this in mind we studied ethylene responses in *Geitlerinema* sp. PCC 7105. This was originally named *Oscillatoria* sp. PCC 7105 (Rippka et al., 1979) and is referred to as Geitlerinema in this paper. This is a filamentous cyanobacterium that binds ethylene and is predicted to contain two ethylene receptors (Wang et al., 2006; Lacey and Binder, 2016). Here we document that ethylene alters phototaxis behavior of Geitlerinema and Synechocystis at low concentrations. These concentrations are below the threshold for most, but not all ethylene responses in plants. However, ethylene dose-binding experiments on Geitlerinema, Synechocystis, and the heterologously expressed ethylene binding domain of SynEtr1 indicate that the affinity of ethylene to the cyanobacterial receptors is similar to what has been reported in plants. Thus, we predict that signal amplification occurs at the level of the receptors.

## Materials and Methods

### Strains and Growth Conditions

*Geitlerinema* PCC 7105 cells were from the laboratory of Anthony Bleecker and were originally obtained from the American Type Culture Collection (stock ATCC29120). *Synechocystis* PCC 6803 cells were obtained from the Pasteur Institute. Liquid cultures of both were maintained in BG-11 medium (Rippka et al., 1979).

### Phototaxis Assays

Phototaxis assays were conducted at 20–21°C in flow-through chambers with continuous gas flow with either ethylene-free air or air with ethylene at the concentrations indicated in each figure. All assays were replicated at least three times.

Phototaxis assays for Synechocystis were conducted for 4 days (d) as previously described using directional white light at a fluence rate of 30 μmol m^–2^ s^–1^ (Lacey and Binder, 2016). These assays were quantified by measuring the maximum distance moved by cells from the leading edge of the colony at the start of the assay. For phototaxis assays on Geitlerinema, cells were placed on 0.4% (v/v) agar BG-11 plates and allowed to grow several days under white fluorescent lights. Efforts were made to start with similar quantities of cells. However, the filamentous nature of this species made it difficult to start with identical numbers of cells. A similar problem has been noted by others studying another filamentous cyanobacteria, *Nostoc punctiforme* (Campbell et al., 2015). The plates were then wrapped with aluminum foil except for a 13 × 13 mm square above the location next to the cells. This area was illuminated from above with white fluorescent lights (42 μmol m^–2^ s^–1^) for 5–7 days to allow cells to move into the illuminated area and grow. At this time, we used one of two methods to examine phototaxis. In some assays, we used methods modified from Biddanda et al. (2015) and illustrated in Supplementary Figure 1A. In this method, the first opening above the cells was blocked, a second opening made 25 mm away, and illumination provided from above. Unless otherwise specified, cells were then allowed to respond to light for 2–5 d in response to 42 μmol m^–2^ s^–1^ of white, or 16 μmol m^–2^ s^–1^ of blue (λ_max_ = 462 nm), green (λ_max_ = 528 nm), or red (λ_max_ = 672 nm) light. In other assays, the Geitlerinema cells were exposed to directional white light using methods modified from Campbell et al. (2015). In this method, each petri dish was masked with black paper except for a 5 mm slit at one edge of the plate. The cells were then exposed for 24 h to directional white light through the slit at a fluence rate of 30 μmol m^–2^ s^–1^. The distance moved toward the light was then quantified by measuring the maximum distance moved by cells from the leading edge of the colony at the start of the assay. For these experiments, white lighting was provided by an LED light panel and monochromatic lighting provided by LED arrays from Quantum Devices Inc. (Barneveld, WI, United States). For both species, images were acquired with a flatbed scanner. In control experiments to examine the effect of these wavelengths of light on growth, we allowed filaments to become established on the BG-11 agar under white light for 1 d, and then exposed the entire plate to either white, red, green, or blue light at the same levels of illumination as used in phototaxis assays. We then scanned the plates 2 d later and used ImageJ to determine the optical density of the colonies.

For polychromatic light experiments, cells were placed on 0.4% (v/v) agar BG-11 plates and exposed to polychromatic light from above the cells for 4 d (Supplementary Figure 1B). Polychromatic light was provided by white light from a slide projector focused onto a prism and images were acquired with a Canon EOS Rebel Xsi.

### Biofilm Assays

Biofilm formation by Synechocystis cells was assayed with modifications to the methods of Agostoni et al. (2016). For this, cells were grown in BG-11 liquid culture to a density of OD_750_ = 0.5 and 15 mL placed into a 250 mL flask. Samples were then incubated in flow through chambers with ethylene-free air or various dosages of ethylene for 5 d under white light (30 μmol m^–2^ s^–1^) provided by an LED panel. Non-adhered cells were removed by aspiration and 0.5% (w/v) crystal violet added for 2 min to stain the cells that remain attached. The stain was removed and the cells washed three times with 15 mL of phosphate-buffered saline. The cells were then resuspended in 10 mL of 95% (v/v) ethanol for 30 min, and the OD_588_ measured. All assays were replicated at least three times.

### RNA Isolation, Complementary DNA Synthesis, and Quantitative Real-Time Reverse Transcriptase (qRT)-PCR

For Synechocystis, cells were exposed to phototaxis conditions for 1 d in ethylene-free air at which time they were either kept in ethylene-free air or treated with ethylene at concentrations ranging from 1 to 1000 nL L^–1^ for 4 h using methods previously described (Lacey et al., 2018). Briefly, ethylene was injected into sealed chambers to yield the designated concentration. Cells were harvested off the agar 4 h later for RNA isolation and further processing. For Geitlerinema, cells were maintained in non-directional light and exposed to ethylene-free air or 1000 nL L^–1^ ethylene for 4 h after which the cells were harvested with forceps. To make harvesting of cells easier, cells were kept in Petri dishes filled with 30 mL BG-11 media. For both species, RNA isolation, complementary DNA synthesis, and qRT-PCR were carried out as previously described (Lacey and Binder, 2016). Primers used for *SynEtr1*, *CsiR1*, *slr1213*, and *slr1214* from Synechocystis have previously been described (Lacey et al., 2018). Synechocystis data were normalized to the *tryptophan synthase gene* (*TrpA*) gene (Zhang et al., 2008) and then to levels of each gene transcript in air-treated controls.

In Geitlerinema, we first determined which housekeeping gene to use. For this, we analyzed the RNA abundance of candidate genes in air- and ethylene-treated cells using qRT-PCR. This was normalized to total RNA (Supplementary Figure 2). From this we determined that the abundance of these transcripts was not significantly altered by application of 1000 nL L^–1^ ethylene. We chose a gene (gene locus WP_026097408) annotated as a *tRNA pseudouridine synthase* (*TruB*) as our housekeeping gene because its levels were very similar in air versus ethylene. Thus, we normalized the RNA abundance of *GeiEtr1* and *GeiEtr2* to the abundance of *TruB* and then to air-treated controls. Primers for qPCR were 5′-ATGTGGGAAACTGTCAAAACTTTATTTT-3′ (forward) and 5′-CCGAAGCCTGCTGGGTAA-3′ (reverse) for *GeiEtr1*, 5′-AT GTGGACCGCTCTCGAATCGCTCC-3′ (forward) and 5′-CCC GAACGAAATCCATGACTGCTGA-3′ (reverse) for *GeiEtr2*, and 5′-ATGGCGGGCTTTCTGAACCTGG-3′ (forward) and 5′-CCGAAAATGGTGTTTGATCGC-3′ for *TruB.*

Quantitative PCR was performed as described in Lacey and Binder (2016). All data represent the average ± SEM from three technical replicates done on three biological replicates.

### Ethylene Binding Assays

Ethylene binding assays were conducted on Geitlerinema and Synechocystis cells as previously described for bacteria (Wang et al., 2006) and on the ethylene binding domain of SynEtr1 fused to glutathione-*S*-transferase (SynEtr1[1-130]GST) expressed in *Pichia pastoris* as described by Lacey and Binder (2016). In control samples, specific ethylene binding at 1000 nL L^–1^ ethylene to Synechocystis cells lacking SynEtr1 (ΔSynEtr1) and *P. pastoris* with empty vector (pPICZ) was determined. The ΔSynEtr1 Synechocystis have been previously described (Lacey and Binder, 2016). Assays were conducted using ^14^C_2_H_4_ custom synthesized by ViTrax (Placentia, CA, United States). Briefly, experiments were conducted on 0.8 (wet weight) of bacteria cells placed on Whatman No. 1 paper filters or 1 g (wet weight) *P. pastoris* cells placed on glass filters. Samples were then treated with either ^14^C_2_H_4_ at the indicated concentrations to determine total binding or ^14^C_2_H_4_ plus 1000-fold excess ^12^C_2_H_4_ to determine non-specific background binding. Specific binding was calculated by subtracting non-specific binding from total binding. All experiments were done in triplicate.

## Results

### Geitlerinema Has Two Putative Ethylene Receptors

The sequenced Geitlerinema genome contains two genes predicted to encode proteins with an ethylene-binding domain (see the Supplementary Data for full DNA and amino acid sequences). We are calling them Geitlerinema *Ethylene response1* (*GeiEtr1*) and *GeiEtr2* following the nomenclature for the first ethylene receptor discovered, *AtETR1* from *Arabidopsis thaliana. GeiEtr1* is at DNA coordinates 3597293–3599260 and is predicted to encode a protein 655 amino acids long, whereas *GeiEtr2* is at DNA coordinates 3147733–3150810 and is predicted to encode a 1025 amino acid long protein. An examination of the genome neighborhoods of these two genes (Supplementary Figure 3) reveals one gene grouped with *GeiEtr1* predicted to encode a lycopene cyclase and two genes grouped with *GeiEtr2* where one is predicted to encode a protein with a diguanylate cyclase domain and the other annotated as a starch phosphorylase.

**FIGURE 1:**
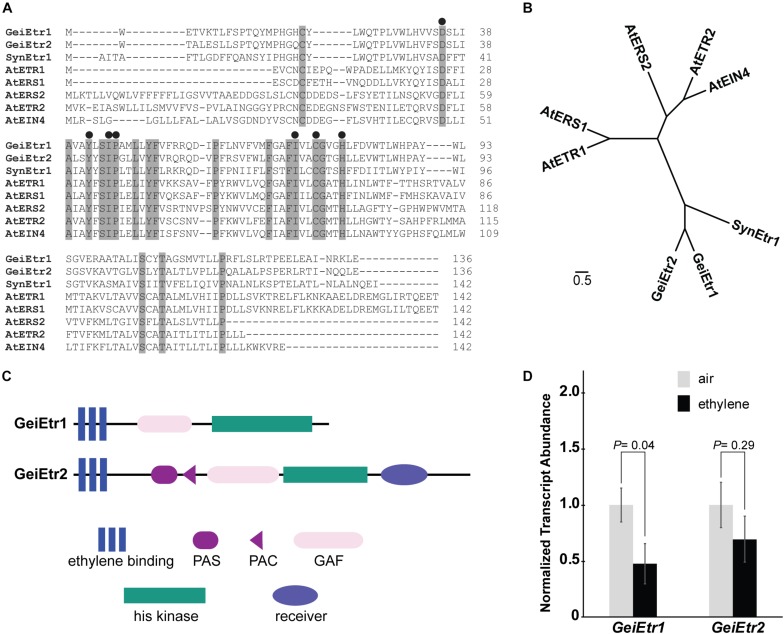
Geitlerinema has two putative ethylene receptors. A BLAST search of the *Geitlerinema* PCC 7105 genome reveals that it has two genes, *GeiEtr1* and *GeiEtr2*, that are predicted to encode proteins with an ethylene binding domain. **(A)** Alignment of the predicted amino acid sequences of the ethylene-binding domains of GeiEtr1 and GeiEtr2 with the ethylene-binding domains of the receptors from *Synechocystis* sp. PCC 6803 (Syn) and *Arabidopsis thaliana* (At). Shaded residues are conserved in all eight sequences. Black circles above the sequences mark amino acids required for ethylene binding to AtETR1. **(B)** Unrooted tree based on the amino acid sequences of the ethylene binding domains from panel **A**. The cladogram was generated using Clustal Omega with default settings and visualized using FigTree version 1.4.2. **(C)** Predicted domain structures of GeiEtr1 and GeiEtr2. Domain predictions were made using the Simple Modular Architecture Research Tool (SMART) http://smart.embl-heidelberg.de/ (Schultz et al., 1998; Letunic et al., 2012). **(D)** Transcript abundance of *GeiEtr1* and *GeiEtr2* in air versus 1000 nL L^–1^ ethylene were determined using qRT-PCR. Data were normalized to the levels of *TruB* and then to the level of each gene in air and represent the average ± SEM. *P*-values were determined using Student’s *t*-test.

The predicted ethylene binding domain of GeiEtr1 shares 43% homology and GeiEtr2 41% homology with the binding domain of the canonical ethylene receptor, AtETR1. An alignment of the ethylene domains from GeiEtr1 and GeiEtr2 with the ethylene receptor from Synechocystis and the five receptors from *A. thaliana* (AtETR1, AtETR2), Ethylene Response Sensor1 (AtERS1), AtERS2, and Ethylene Insensitive4 (AtEIN4) reveals that both proteins from Geitlerinema have retained many amino acids in common with functional ethylene receptors. Seven amino acid residues are required for ethylene binding to AtETR1 (Rodriguez et al., 1999; Wang et al., 2006). All seven of these amino acids are conserved in GeiEtr1 and GeiEtr2 suggesting that they too can bind ethylene (Figure 1A). Consistent with previous research (Hua et al., 1998), this alignment also shows that the subfamily 1 receptors from *A. thaliana*, AtETR1 and AtERS1, have a short N-terminus extension (approximately 19 amino acids) ahead of the ethylene binding domain, whereas there is a longer hydrophobic stretch of amino acids (approximately 50 amino acids) in the subfamily 2 receptors, AtERS2, AtETR2, and AtEIN4. The proteins from cyanobacteria have an N-terminal stretch intermediate in length (approximately 30 amino acids) between the subfamily 1 and 2 receptors. It is also interesting to note that whereas the plant receptors have two cysteines near the N-terminus that form disulfide bonds to form stable homodimers (Schaller et al., 1995; Hall et al., 2000), the cyanobacteria proteins only have one cysteine in this region of the protein that may fulfill the same function. An unrooted cladogram based on the amino acid sequences of the ethylene binding domains of these seven proteins shows that the ethylene receptors from plants fall into two subfamilies with the bacterial receptors forming a distinct third subfamily (Figure 1B), consistent with a previous analysis (Wang et al., 2006). The predicted domain structure of GeiEtr1 is similar to AtERS1 and AtERS2 where there is a N-terminal ethylene binding domain followed by a GAF (for cGMP-specific phosphodiesterase, adenyl cyclases, and FhlA) domain and a C-terminal kinase domain, but no receiver domain (Figure 1C). By contrast, GeiEtr2 has additional domains with a PAS (for Per-Arnt-Sim) and PAC domain between the ethylene binding and GAF domains as well as a C-terminal receiver domain. The arrangement of PAS, PAC, and GAF domains in GeiEtr2 is reminiscent of the domain arrangement of SynEtr1 that functions as a photoreceptor.

We were curious to know if either receptor is expressed and whether or not ethylene affected the transcript abundance of either gene. To answer these questions we extracted RNA from samples kept in air versus 1000 nL L^–1^ ethylene for 4 h. We chose this dosage of ethylene because it is commonly used in plant research and has been shown to affect the physiology and growth of Synechocystis (Lacey and Binder, 2016; Henry et al., 2017) and causes wide-spread changes in the transcriptome of Synechocystis (Lacey et al., 2018). Also, we have previously found that 1000 nL L^–1^ ethylene alters transcript abundance of various genes in Synechocystis cells within 4 h (Lacey et al., 2018). From this analysis we observed that both *GeiEtr1* and *GeiEtr2* are expressed in air. Upon application of ethylene, *GeiEtr1* abundance decreased, but the abundance of *GeiEtr2* showed no statistically significant change (Figure 1D).

### Ethylene Alters Phototaxis of Geitlerinema

**FIGURE 2:**
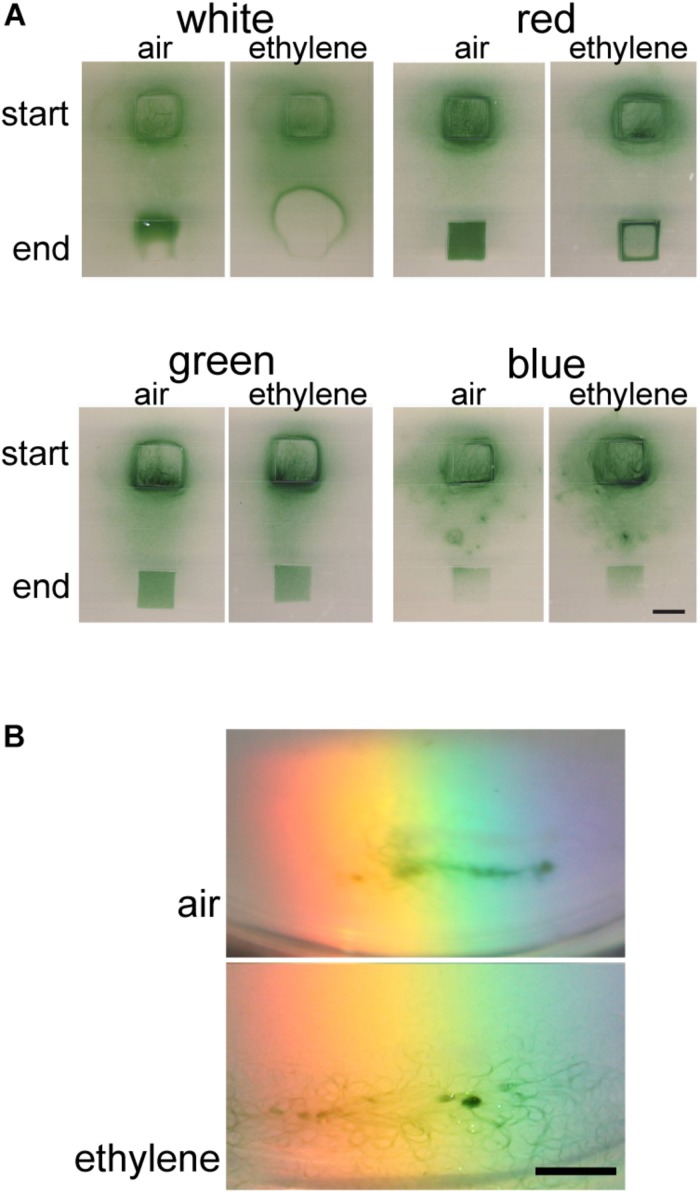
Ethylene alters phototaxis of Geitlerinema. Cells were placed on soft agar and exposed to light as described in the section “Materials and Methods.” Phototaxis in air versus 1000 nL L^–1^ exogenous ethylene was examined. **(A)** Phototaxis responses to 42 μmol m^–2^ s^–1^ of white light for 5 d compared to 16 μmol m^–2^ s^–1^ of red light for 2 d, green light for 2 d, or blue light for 5 d are shown. The position where the colony of cells started and end position of lighting is indicated. Scale bar = 1 cm. **(B)** Response of cells to polychromatic light for 5 d. Scale bar = 0.5 cm.

We have previously shown that ethylene accelerates phototaxis of Synechocystis toward white light (Lacey and Binder, 2016). We therefore examined the effect of ethylene on phototaxis of Geitlerinema. Because this is a filamentous cyanobacterium, we adapted the methods of Biddanda et al. (2015) to conduct these assays where the bacteria were exposed to an area of illumination at a distance from their starting location (Supplementary Figure 1A). In air, cells displayed phototaxis movement in response to white light where most, but not all cells, moved to the new position of illumination after 5 d (Supplementary Figure 4). Given this result we conducted phototaxis assays toward white light for 5 d to determine whether application of ethylene increased or decreased movement. Interestingly, the application of 1000 nL L^–1^ ethylene altered the response to white light so that the cells formed a ring outside the area of illumination (Figure 2A). To determine if this ring of cells is due to higher light sensitivity causing the cells to avoid the area with the highest light levels, we conducted these assays at a 10-fold dimmer light intensity. At this dimmer light level, the cells move into the entire area of illumination so that no ring of cells is present (Supplementary Figure 5) suggesting that ethylene sensitizes Geitlerinema to higher light intensities.

We have also previously demonstrated that ethylene accelerates phototaxis of Synechocystis toward monochromatic light including red, green, and blue light (Lacey and Binder, 2016). Therefore, we examined phototaxis of Geitlerinema cells in response to these colors of light. In air, phototaxis occurred faster in response to red and green light compared to white light (Supplementary Figure 4). By contrast, the cells did not phototaxis in response to blue light and appeared to show unbiased movement with few cells accumulating in the area of illumination. This is consistent with results using Synechocystis where blue light normally does not cause phototaxis and has been found to promote growth and inhibit motility (Wilde et al., 2002; Chau et al., 2017). We also examined the effects of these different light qualities on cell growth and found that growth occurred in white, red, and green light at similar rates but growth did not occur in blue light (Supplementary Figure 6). This suggests that the larger number of cells in the new area of illumination in red and green light is not simply caused by more growth compared to white light.

Because red and green light cause faster phototaxis compared to white light, we shortened the assay time-frame from 5 to 2 d in order to determine whether ethylene stimulates or inhibits phototaxis. Under these conditions, 1000 nL L^–1^ ethylene altered the phototaxis pattern in response to red light where the cells aggregated at the edge of the area of illumination (Figure 2A). By contrast, ethylene caused no measurable change in phototaxis in response to either green or blue light. This is in contrast to our observations with Synechocystis where 1000 nL L^–1^ ethylene caused a measurable increase in phototaxis toward both (Lacey and Binder, 2016).

Cyanobacteria integrate various light inputs including wavelength information (Chau et al., 2017). We were therefore curious to examine the response of Geitlerinema cells to polychromatic light. In air, the cells moved into the light spectrum between orange and blue (Figure 2B). By contrast, in the presence of 1000 nL L^–1^ ethylene the cells moved into a wider range of wavelengths of light that included red light. Together, these results indicate that ethylene affects the phototaxis response of Geitlerinema cells to different wavelengths of light.

### Low Levels of Exogenous Ethylene Affect Geitlerinema

**FIGURE 3:**
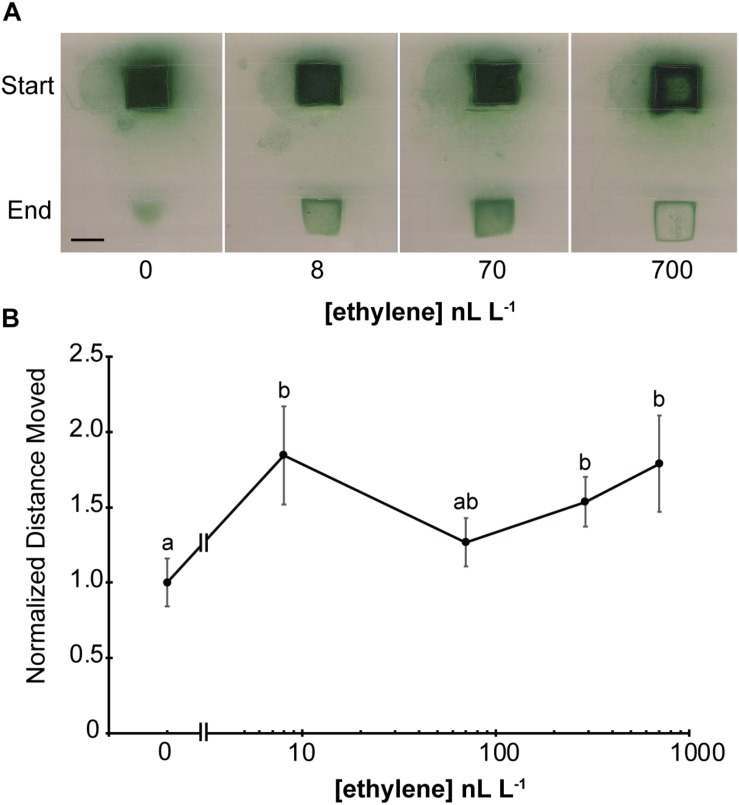
Geitlerinema responds to low levels of exogenous ethylene. **(A)** Cells were placed on soft agar and exposed to white light from above using a protocol modified from Biddanda et al. (2015) as described in the section “Materials and Methods.” The position where the colony of cells started and end position after 5 d of lighting are indicated. Scale bar = 1 cm. **(B)** Phototaxis assays were conducted by exposing cells to directional light for 24 h using a protocol modified from Campbell et al. (2015) as described in the section “Materials and Methods.” The maximum distance moved from the initial colony position was measured and normalized to distance moved in air. Data represent the average ± SEM from five replicates. Statistical analysis was done with ANOVA and the different letters indicate significant differences (*P* < 0.05). In both panels, cells were kept in flow-through chambers maintained at the indicated concentration of exogenous ethylene.

Plants respond to a wide range of ethylene concentrations and even show transient growth inhibition at very low concentrations (0.2 nL L^–1^) of exogenous ethylene (Chen and Bleecker, 1995; Binder et al., 2004). We therefore wished to know the threshold concentration of ethylene that affects phototaxis of Geitlerinema. To examine this we conducted phototaxis assays in response to white light for 5 d in the presence of different concentrations of ethylene or ethylene-free air (Figure 3A and Supplementary Figure 7). Under these conditions, even the lowest concentration used, 8 nL L^–1^, resulted in faster phototaxis since more filaments of cells moved into the illuminated area of the plate than were observed in ethylene-free air. Increasing the ethylene levels to 70 nL L^–1^ caused more phototaxis. By contrast, treatment with 700 nL L^–1^ ethylene caused the cells to form a distinct band around the edge of the illuminated area reminiscent to the ring that formed farther from the light with 1000 nL L^–1^ (Figure 2).

To gain a better understanding about what appears to be enhanced phototaxis at low levels of ethylene, we also conducted phototaxis experiments where the cells were exposed to directional white light. In air, cells moved from the original colony in all directions relative to the light, but the largest distance moved was toward the light source (Supplementary Figure 8). Application of ethylene at the lowest dose used (8 nL L^–1^) significantly increased the distance moved by cells toward directional white light and the response seems to be saturated by this level of ethylene (Figure 3B and Supplementary Figure 8). These data support the idea that low ethylene levels increase phototaxis in this cyanobacterium.

### Low Levels of Exogenous Ethylene Affect Synechocystis

**FIGURE 4:**
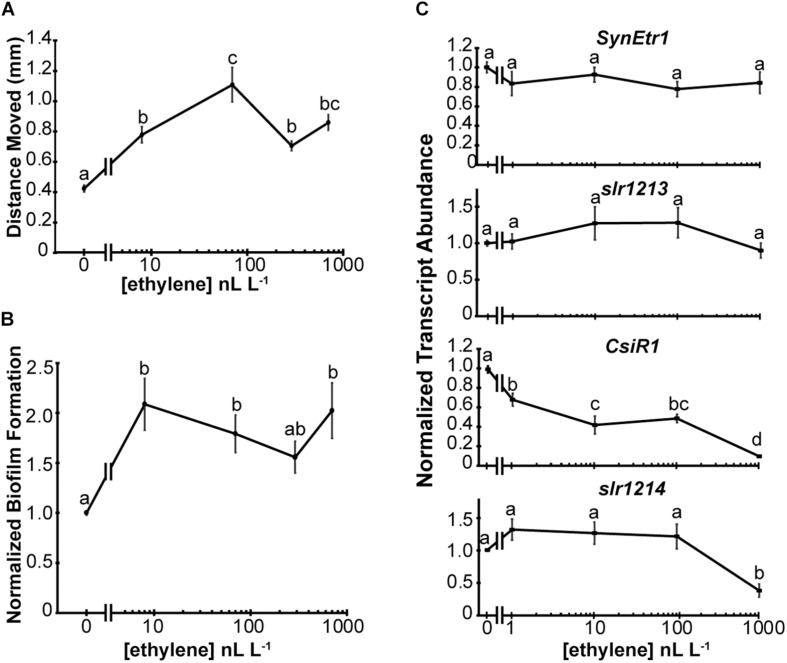
Synechocystis responds to low levels of exogenous ethylene. The effects of various dosages of exogenous ethylene on Synechocystis were measured. **(A)** Phototaxis assays were conducted and the maximum distance moved from the initial colony position measured. **(B)** Biofilm formation was quantified by measuring the staining of attached cells using Crystal Violet. Data are normalized to staining of cells in the absence of ethylene. In panels **(A,B)**, cells were kept in flow-through chambers maintained at 0, 8, 70, 290, or 700 nL L^–1^ exogenous ethylene for 4 d and data are the average ± SEM. **(C)** The gene transcript abundance of *SynEtr1*, *slr1213*, *CsiR1*, and *slr1214* was measured using qRT-PCR from RNA extracted from cells maintained at 0, 1, 10, 100, or 1000 nL L^–1^ exogenous ethylene for 4 h in sealed chambers under phototaxis conditions. Data were normalized to the transcript levels of the *TrpA* reference gene and normalized to cells kept in ethylene-free air. Data represent the average ± SEM from three biological replicates with three technical replicates each. In all panels, statistical analyses were done with ANOVA and the different letters indicate significant differences (*P* < 0.05).

Because low levels of exogenous ethylene affect Geitlerinema, we tested whether or not low levels of ethylene also affect the physiology of Synechocystis. For this we measured phototaxis and biofilm formation, both of which are increased by application of 1000 nL L^–1^ ethylene (Lacey and Binder, 2016). Application of ethylene at the lowest dose used (8 nL L^–1^) significantly increased the distance moved by cells in response to directional white light (Figure 4A). There is a slight increase in phototaxis at 70 nL L^–1^ but the response seems to be largely saturated with 8 nL L^–1^ applied ethylene. A similar dose–response curve was observed with biofilm formation except that saturation of the response at 8 nL L^–1^ is more clearly seen (Figure 4B). As a comparison, the threshold ethylene concentration typically observed for responses in plants is above 10 nL L^–1^ with saturation of responses occurring in a concentration range of 1–100 μL L^–1^ ethylene depending on the response being measured and the species studied (Chadwick and Burg, 1970; Lyon, 1970; Burdett, 1972; Goeschl and Kays, 1975; De Munk and Duineveld, 1986; Beaudry and Kays, 1988; Chen and Bleecker, 1995).

These assays were conducted using flow-through chambers to maintain constant O_2_ and CO_2_ concentrations over the long time period (4 d) of the assays. A limitation of this method is that it is difficult to reliably deliver lower ethylene concentrations. Previously, we showed that application of 1000 nL L^–1^ ethylene causes a rapid (within 30 min) decrease in the transcript levels of *CsiR1* and *slr1214* (Lacey et al., 2018). This allowed us to conduct shorter term experiments where ethylene was simply injected into a sealed chamber with phototaxing cells. We compared the transcript levels of *SynEtr1*, *slr1213*, *CsiR1*, and *slr1214* after 4 h treatments with varying dosages of exogenous ethylene. Results from this showed that ethylene at dosages between 1 and 1000 nL L^–1^ had no significant effect on the transcript abundance of either *SynEtr1* or *slr1213* (Figure 4C). By contrast, *CsiR1* transcript abundance was altered by application of as low as 1 nL L^–1^ ethylene and increasing levels of ethylene resulted in a concomitant decrease in the levels of *CsiR1* transcript. By contrast, lower dosages of exogenous ethylene had no effect on *slr1214* transcript. However, treatment with 1000 nL L^–1^ ethylene resulted in a decrease in *slr1214.* These results with 1000 nL L^–1^ ethylene are consistent with our prior results examining these four transcripts (Lacey et al., 2018). Together, these data indicate that Synechocystis responds to ethylene at levels as low as 1 nL L^–1^.

### Ethylene Dose-Binding to Geitlerinema, Synechocystis, and SynEtr1[1-130]GST

**FIGURE 5:**
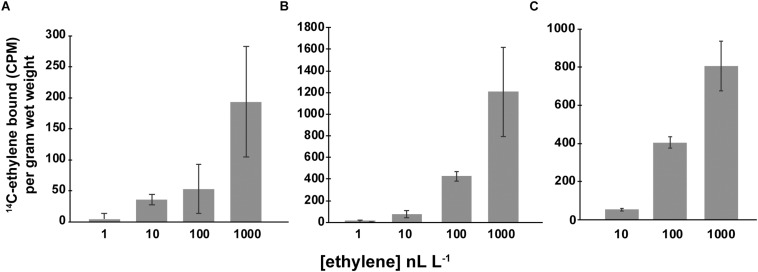
Specific ethylene binding. Ethylene-binding assays were conducted as detailed in the section “Materials and Methods” on **(A)** Geitlerinema cells, **(B)** Synechocystis cells, and **(C)** *P. pastoris* cells expressing the ethylene binding domain of SynEtr1 (SynEtr1[1-130]GST). Assays were carried out at the indicated ethylene concentrations of ^14^C-ethylene to determine total binding. Non-specific binding was determined using the same concentrations of ^14^C-ethylene in the presence of 1000-fold excess non-radioactive ethylene. Specific binding was then calculated by subtracting non-specific binding activity from total binding activity. Data are the average specific binding ± SD.

The ethylene dose-dependency of binding to AtETR1 parallels the dose-dependency for long-term growth inhibition of dark-grown *A. thaliana* seedlings in response to ethylene (Chen and Bleecker, 1995; Schaller and Bleecker, 1995). This suggests that this response is related to the binding affinity to the receptors. We were curious to know if a similar relationship between ethylene binding and responses in Synechocystis and Geitlerinema existed. If true, we predicted that ethylene dose-binding characteristics should saturate at around 10–100 nL L^–1^ ethylene. To test this, we conducted ethylene dose-binding experiments across a range of ethylene concentrations on Geitlerinema and Synechocystis cells, and SynEtr1[1-130]GST expressed in *P. pastoris* (Figure 5). In all three cases, ethylene binding continued to increase as ethylene levels were increased up to 1000 nL L^–1^, demonstrating that ethylene-binding activity does not saturate at 100 nL L^–1^. At 1000 nL L^–1^, there was no specific ethylene-binding activity detected in Δ*SynEtr1* Synechocystis cells with *SynEtr1* deleted or in *P. pastoris* cells that were expressing empty vector, consistent with what has previously been reported (Rodriguez et al., 1999; McDaniel and Binder, 2012). Ethylene binding did not reach obvious saturation in the range of concentrations we tested so we estimated the *K*_*d*_-values with curve fitting. We fitted the data with GraphPad Prism (Ver. 7.04) using default settings for one binding site and measuring specific binding. This yielded sigmoidal curves with estimated *K*_*d*_-values of 335 ± 227 nL L^–1^ for Geitlerinema, 248 ± 16 nL L^–1^ for Synechocystis, and 127 ± 12 nL L^–1^ for SynEtr1[1-130]GST. These values are in the same range as what has been observed in many plants (Sisler, 1979; Goren and Sisler, 1986; Sisler et al., 1986; Smith et al., 1987; Blankenship and Sisler, 1989, 1993; Sanders et al., 1990) and somewhat higher than what has been reported for *A. thaliana* plants (Sanders et al., 1991) and exogenously expressed AtETR1 (Schaller and Bleecker, 1995). This suggests that the sensitivity of ethylene responses in these cells is not explained simply by the binding affinity of ethylene to the receptors.

## Discussion

We have previously shown that the cyanobacterium Synechocystis contains a functional ethylene receptor that regulates cell physiology including phototaxis and biofilm formation and that many other bacteria may contain functional ethylene receptors (Rodriguez et al., 1999; Wang et al., 2006; Lacey and Binder, 2016). In this study we provide evidence supporting the idea that another cyanobacterium, Geitlerinema, responds to ethylene and contains two ethylene receptor isoforms. Application of ethylene to Geitlerinema alters phototaxis indicating a conservation of function for ethylene signaling in both Geitlerinema and Synechocystis. At low ethylene concentrations, ethylene stimulates phototaxis toward white light for both species. It is unclear if this is occurring because of faster movement of individual cells/filaments or movement that is more directed toward the light or both. Using single-cell tracking assays, Kuchmina et al. (2017) demonstrated that higher concentrations of endogenously produced ethylene do not affect the speed of cells, but rather cause movement that is more directed toward the illumination (Kuchmina et al., 2017). It is unknown if this also applies to Synechocystis cells once they aggregate as used in our assays or to Geitlerinema filaments. At higher concentrations, ethylene appeared to sensitize Geitlerinema to the light so that the cells tended to avoid higher levels of illumination. No such sensitization caused by ethylene has been observed in Synechocystis. Like Synechocystis (Lacey and Binder, 2016), ethylene alters phototaxis of Geitlerinema in response to white light and monochromatic red light. However, unlike our prior results studying Synechocystis, ethylene does not have a measurable effect on phototaxis of Geitlerinema in response to monochromatic blue or green light. Interestingly, ethylene affected responses to polychromatic light where Geitlerinema cells phototaxed into a wider range of wavelengths of light in the presence of ethylene versus in ethylene-free air. This shows that ethylene can affect wavelength integration by these cells.

A model for signal transduction from SynEtr1 in Synechocystis has developed (Ramakrishnan and Tabor, 2016) where UV-A light stimulates histidine autophosphorylation of SynEtr1 followed by phosphotransfer to a conserved aspartate on slr1213. The phosphorylated slr1213 enhances the transcription of *CsiR1* and *slr1214* which contain a common transcription start site and appears to be co-transcribed in response to UV-A light (Ramakrishnan and Tabor, 2016). In contrast to UV-A light, application of 1000 nL L^–1^ ethylene reduces the transcript abundance of *CsiR1* and *slr1214* (Lacey et al., 2018). It is currently unknown whether or not ethylene signaling from SynEtr1 also affects histidine kinase activity to regulate *CsiR1* and *slr1214* levels. However, application of ethylene reveals that the regulation of *CsiR1* and *slr1214* is not entirely overlapping. First, we previously showed that application of 1000 nL L^–1^ ethylene caused a rapid and prolonged down regulation of *CsiR1*, whereas this treatment caused a rapid and transient down-regulation of *slr1214* (Lacey et al., 2018). In this study we provide further evidence for more complex regulation where low levels of ethylene decrease *CsiR1* levels, but it requires a much higher level of ethylene to reduce the abundance of *slr1214*. We currently do not know the basis for these differences in regulation but it is possible that the transcript stabilities of these genes are regulated differently.

In Synechocystis, the ethylene dose–responses for phototaxis and biofilm formation show an inverse correlation with the dose–response for *CsiR1* abundance suggesting that *CsiR1* plays an important inhibitory role for these two responses. However, its function in Synechocystis and the mechanisms by which it affects Synechocystis physiology are poorly studied. One possibility is that *CsiR1* is functioning in transcriptional or post-transcriptional regulation in Synechocystis (Georg et al., 2009; Hernández-Prieto et al., 2012). In Synechocystis, both biofilm formation and motility are dependent on type IV pili (Bhaya et al., 1999, 2000; Yoshihara et al., 2001; Burriesci and Bhaya, 2008; Schuergers et al., 2015). The RNA chaperone Hfq affects Synechocystis motility and type IV pilus function (Dienst et al., 2008; Schuergers et al., 2014) suggesting that *CsiR1* may also affect motility and biofilm formation by altering type IV pili, perhaps by altering expression of certain pilin proteins. However, it is likely that regulation by ethylene is more complex than simply regulating *CsiR1* since removing slr1214 eliminates physiological responses to ethylene (Lacey and Binder, 2016).

We have previously studied the effects of ethylene on Synechocystis using 300 and 1000 nL L^–1^ exogenous ethylene (Lacey and Binder, 2016; Henry et al., 2017; Lacey et al., 2018). This is a common concentration range to use in plants, but in aqueous environments, ethylene levels are often reported to be lower than this (Abeles et al., 1992). We therefore examined the physiological responses of Synechocystis and Geitlerinema to a range of ethylene concentrations and found that both species physiologically respond to levels of ethylene as low as 8 nL L^–1^. We quantified the effects of ethylene on increasing biofilm formation and phototaxis in Synechocystis and discovered that both responses saturate at between 8 and 70 nL L^–1^. This suggests that both responses occur over a narrow dynamic range of ethylene concentrations, although we cannot rule out that these cells have physiological responses that occur at ethylene concentrations lower than used in this study. By comparison, plants have a wide dynamic range over several orders of magnitude of ethylene concentration. For instance, the growth of dark-grown *A. thaliana* seedlings is inhibited in a dose-dependent manner over a range of ethylene concentrations between 10 and 1000 nL L^–1^ (Chen and Bleecker, 1995). It is interesting to note that induction of chitinase-B also has dose-dependent induction over two orders of magnitude ethylene concentration. However, whereas growth inhibition saturates at 1000 nL L^–1^, chitinase-B induction saturates at a 10-fold higher concentration (Chen and Bleecker, 1995). It is therefore of note that the reduction of *CsiR1* transcript abundance in Synechocystis occurs in a dose-dependent manner over a wide range of ethylene concentrations between 1 and 1000 nL L^–1^. This raises the possibility that there may be other physiological responses in Synechocystis occurring in different ranges of ethylene concentration from biofilm formation and phototaxis. It is also interesting that *CsiR1* transcript abundance was reduced by application of as low as 1 nL L^–1^ ethylene. This low level of ethylene is below the threshold for most responses reported in plants (Abeles et al., 1992). Exceptions are that stimulation of *Ricinodendron rautanenii* seed germination has been observed with 1 nL L^–1^ treatments (Keegan et al., 1989) and transient growth inhibition responses occur in dark grown *A. thaliana* seedlings down to 0.2 nL L^–1^ ethylene (Binder et al., 2004).

The long-term growth inhibition response of dark-grown *A. thaliana* to ethylene has a dose-dependency that parallels the dose-dependency of ethylene binding to AtETR1 (Chen and Bleecker, 1995; Schaller and Bleecker, 1995). This led us to predict that ethylene binding to Geitlerinema and Synechocystis cells, as well as heterologously expressed SynEtr1, would saturate at between 8 and 100 nL L^–1^ ethylene. However, our results indicate that this is not the case. Thus, unlike the long-term growth inhibition response of dark-grown *A. thaliana* seedlings to ethylene, there is no obvious correlation between the amount of ethylene bound and physiological responses in these two species of cyanobacteria. It is interesting to note that the dose-dependency of ethylene binding to Synechocystis and SynEtr1[1-130]GST parallels the decrease in *CsiR1* transcript caused by ethylene. The *K*_d_ for ethylene binding to AtETR1 heterologously expressed in yeast is reported at 36 nL L^–1^ (Schaller and Bleecker, 1995) yet *A. thaliana* seedlings are able to respond to ethylene concentrations approximately 300-fold below this level with a transient growth inhibition response (Binder et al., 2004). Our results indicate there may be a similar discrepancy between ethylene binding and ethylene responses where Synechocystis responds to 1 nL L^–1^ ethylene yet the estimated binding affinity is over 100-fold higher. These observations suggest that signal amplification is occurring. In bacterial chemotaxis receptors, such amplification occurs because of physical clustering of the receptors. In this model, binding of ligand to one receptor causes conformational changes in surrounding, ligand-free receptors through physical interactions (Briegel and Jensen, 2017; Bi and Sourjik, 2018). Thus, it is possible that amplification of ethylene signaling in these cyanobacteria, as well as in plants, is occurring at the level of the receptors. However, it remains to be determined whether a similar clustering of ethylene receptors and amplification of signal is occurring in either plants or cyanobacteria.

The ecophysiological role of ethylene for cyanobacteria remains unanswered but it is likely that cyanobacteria encounter ethylene in the environment produced by other organisms or produced abiotically from sunlight photochemically converting dissolved organics to ethylene (Swinnerton and Linnenborn, 1967; Wilson et al., 1970; Swinnerton and Lamontagne, 1974; Ratte et al., 1993, 1998). It is known that ethylene diffuses in water and ethylene levels vary in aqueous environments depending on environmental conditions resulting in ethylene concentrations in the range where we observe responses (Swinnerton and Lamontagne, 1974; Plass et al., 1992; Ratte et al., 1993, 1998). Thus, ethylene may be acting as a signal for cells to move into light conditions that optimize photosynthesis or reduce light stress or it may be a signal to establish a symbiotic relationship with another organisms. Our results showing that ethylene causes Geitlerinema cells to avoid high light supports the idea that it is a stress signal. However, these are not mutually exclusive ideas and ethylene may be regulating a variety of functions.

In summary, we have shown that two cyanobacteria species respond to low levels of ethylene. Our results support the hypothesis that ethylene perception evolved prior to green plants and is likely to be wide-spread in cyanobacteria species. Given the diversity of putative receptors in various microbes and the presence of multiple receptor isoforms in certain species (Mount and Chang, 2002; Wang et al., 2006; Lacey and Binder, 2016; Hérivaux et al., 2017; Kabbara et al., 2018; Papon and Binder, 2019), it is likely that the mechanisms of ethylene signaling and responses controlled by ethylene are diverse.

## Data Availability

All datasets for this study are included in the manuscript and/or the Supplementary Files.

## Author Contributions

RL, CA, and BB designed the experiments. BB wrote the manuscript with the help of RL. All authors performed the experiments.

## Conflict of Interest Statement

The authors declare that the research was conducted in the absence of any commercial or financial relationships that could be construed as a potential conflict of interest.
