# Supplementary material for: Cyanobacteria Respond to Low Levels of Ethylene
Source: Front Plant Sci. 2019 Jul 30;10:950. doi: 10.3389/fpls.2019.00950 (PMC6682694; doi:10.3389/fpls.2019.00950)
Supplement: Supplementary file 1 [file Image_1.PDF]

Supplemental Figures

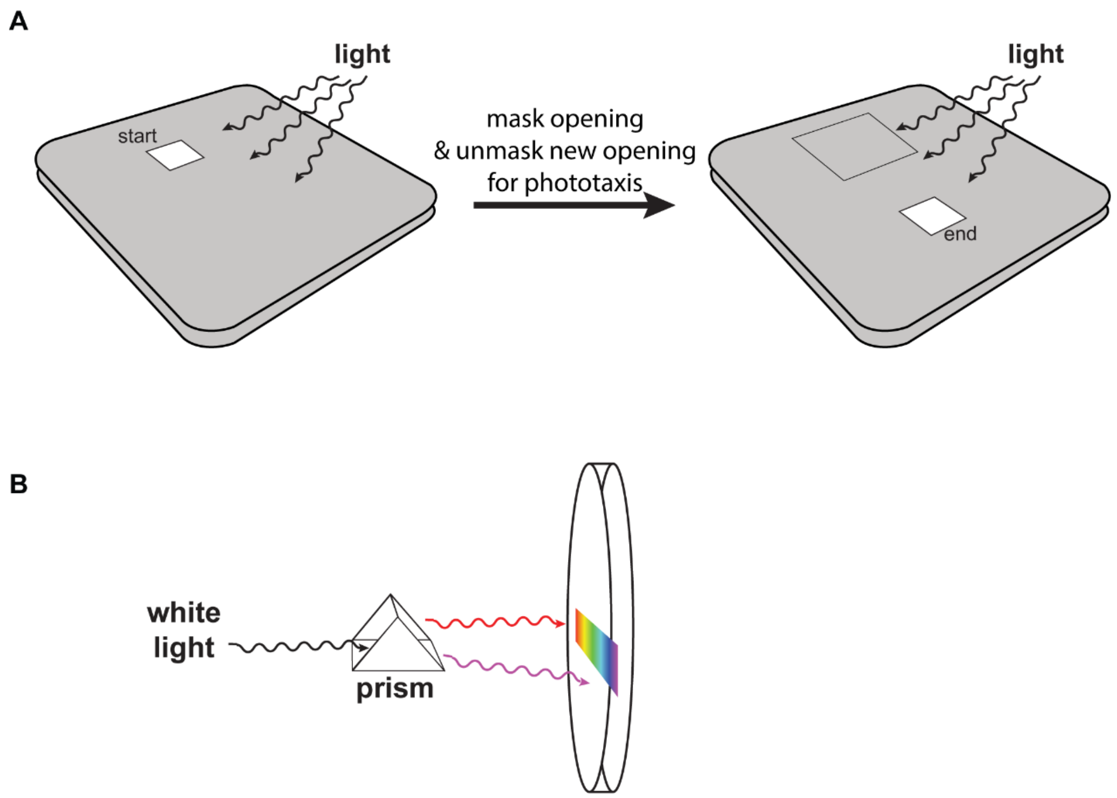

Supplemental Figure 1. Diagrams of phototaxis assay for Geitlerinema cells. (A) For monochromatic and white light experiments cells were exposed from above. Plates were masked with aluminum foil except for a square (start) that was exposed to white light to allow cells to move into and grow in this area. This square was then masked, and a new square was unmasked 25 mm away. Cells were then exposed to either white light, monochromatic blue light, monochromatic red light, or monochromatic green light. **(B)** For polychromatic light experiments, cells were exposed to polychromatic light generated by shining white light from a slide projector through a prism.

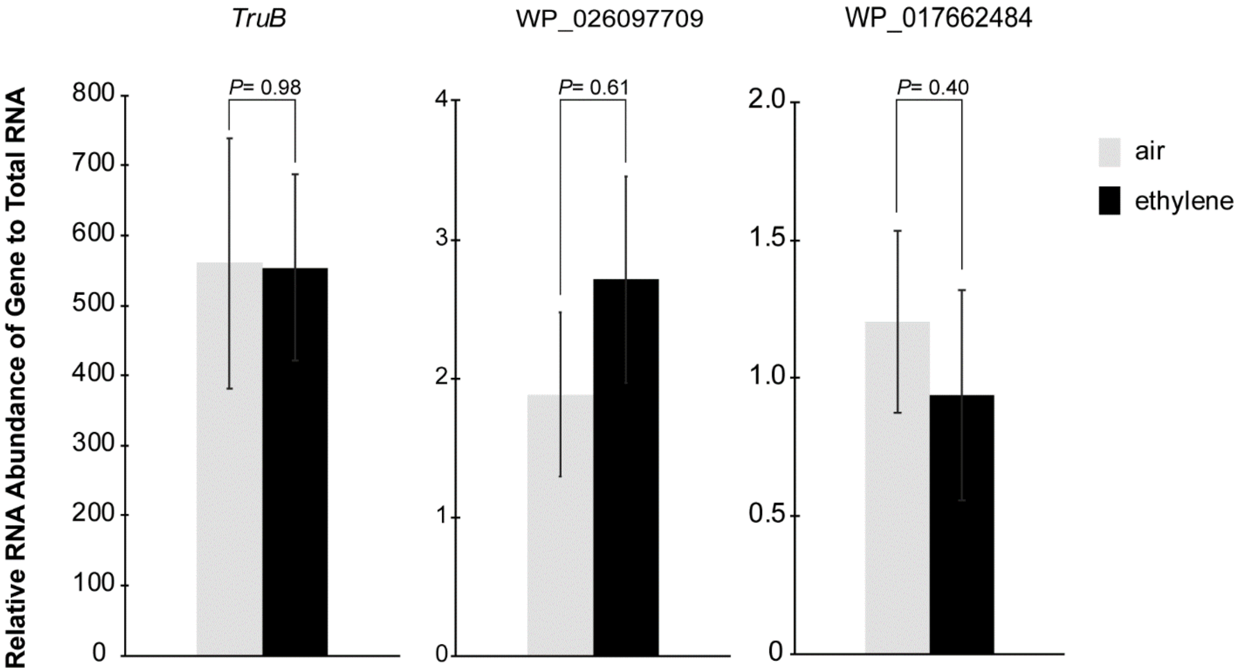

Supplemental Figure 2. RNA abundance relative to total RNA for selected genes. The RNA abundance of several potential housekeeping genes in Geitlerinema were evaluated for responses to ethylene using qRT-PCR. The cycle threshold (Ct) value was converted to a 2^Ct value and then normalized to total RNA in the sample. Data represents the average ± SEM of two biological replicates with three technical replicates for each biological replicate. The genes examined and primers used were:

gene locus WP_026097408 annotated as a tRNA pseudouridine synthase (*TruB*)
Forward 5'-atggcgggctttctgaacctgg-3', Reverse 5'-ccgaaaatggtgtttgatcgc-3'

gene locus WP_026097709 annotated as a threonine synthase
Forward 5'-atgacccaggcgattgaacgcc-3', Reverse 5'-ctggaatggcagcaggtgctgg-3'

gene locus WP_017662484 annotated as a DEAD/DEAH box helicase
Forward 5'-atggcgattctgcatggcagctg-3', Reverse 5'-agcgcgattgtggaagaagatgaagtg-3'

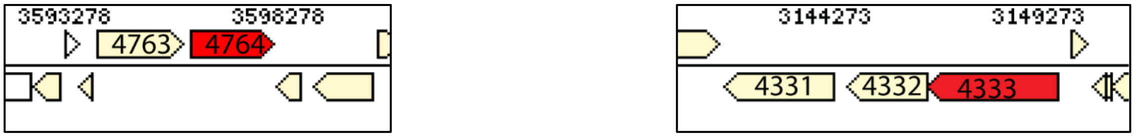

Supplemental Figure 3. Gene Neighborhood Information for *GeiEtr1* (left) and *GeiEtr2* (right). Red marks *GeiEtr1* (4764) and *GeiEtr2* (4333). Gene 4763 next to *GeiEtr1* encodes for a predicted lycopene cyclase. Next to *GeiEtr2*, gene 4332 encodes for a protein predicted to contain a diguanylate cyclase domain and gene 4331 for a putative starch phosphorylase. From https://img.jgi.doe.gov/

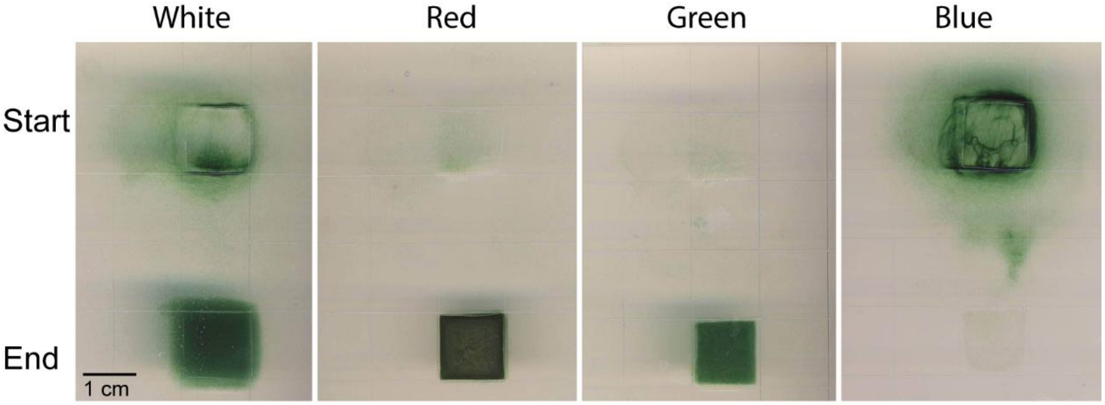

Supplemental Figure 4. Phototaxis of Geitlerinema cells in air for 5 days. Assays were conducted as described in the materials and methods. The position where the colony of cells started and end position of illumination are indicated.

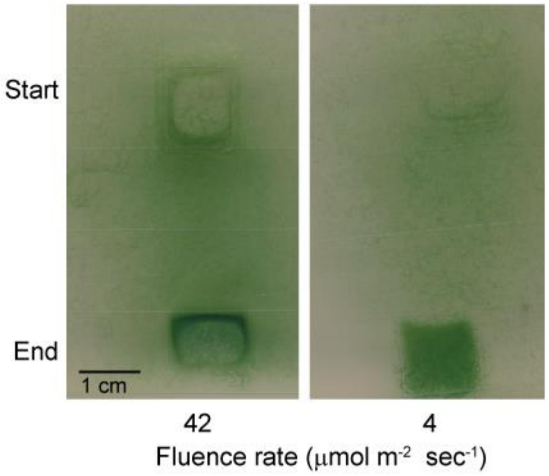

Supplemental Figure 5. Effect of ethylene on phototaxis of Geitlerinema cells at different light fluence rates. Phototaxis assays were conducted under white light at the indicated fluence rates for 3 days in the presence of 1000 nL L^{-1} ethylene as described in the materials and methods.

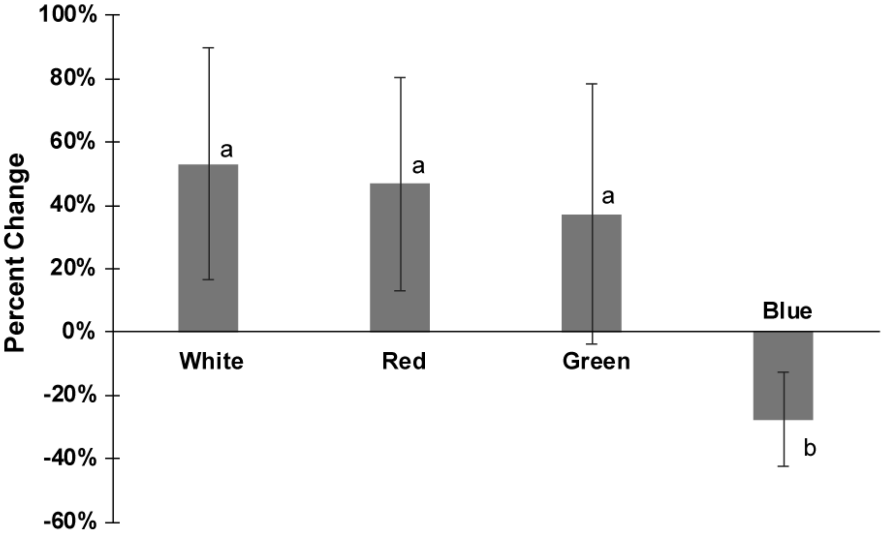

Supplemental Figure 6. Growth of Geitlerinema colonies in different light. Assays were conducted as described in the materials and methods where colonies of filaments were allowed to grow on BG-11 agar plates for 2d under the indicated colored light. Images of the colonies were obtained at the start and end of the growth period and ImageJ used to determine the density of colonies. Data is the average ± SD from 3 samples. Statistical analysis was done with ANOVA and the different letters indicate significant differences ($P < 0.05$).

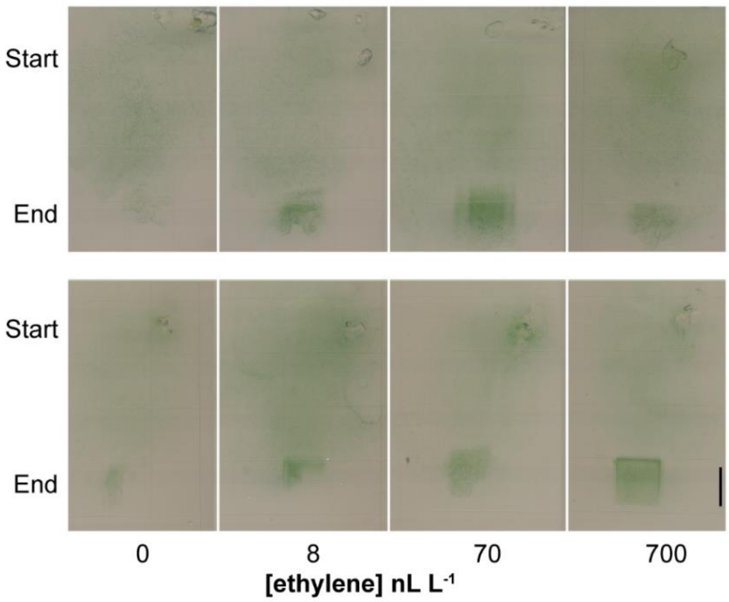

Supplemental Figure 7. Additional experiments showing that ethylene affects Geitlerinema phototaxis. Two additional phototaxis assays were conducted under similar conditions as used in figure 3A with overhead lighting at the indicated concentrations of ethylene. Scale bars = 1 cm.

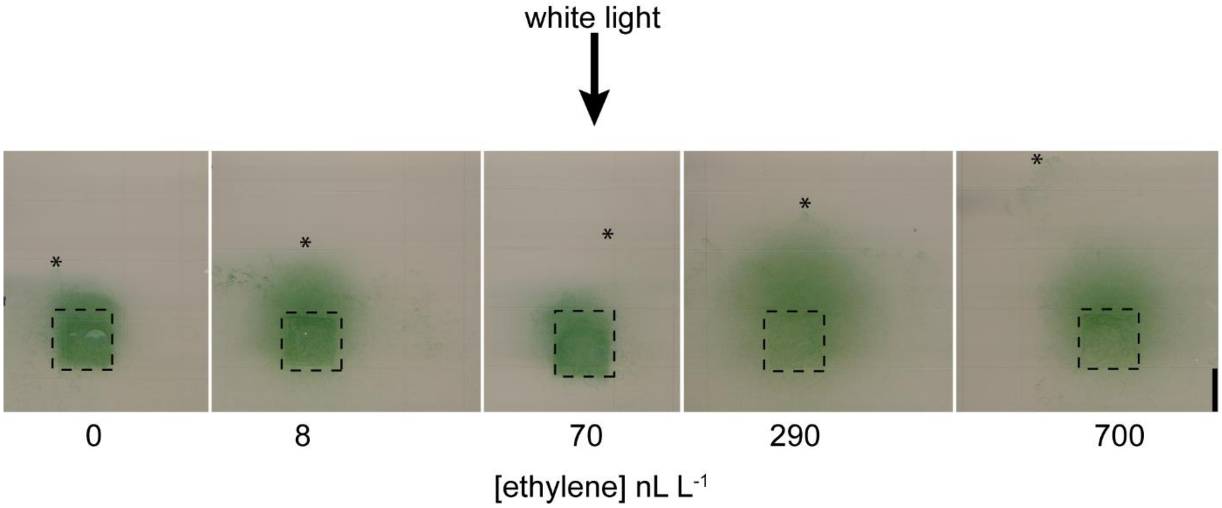

Supplemental Figure 8. Ethylene affects the phototaxis response of Geitlerinema. Colonies of Geitlerinema cells were spotted on soft agar plates and exposed to directional white light as described in the materials and methods. The dotted box outlines the starting position of the colony of cells and the asterix marks the location of farthest movement towards the light. Scale bar = 1 cm.
