# Supplementary material for: Cyanobacteria Respond to Low Levels of Ethylene
Source: Front Plant Sci. 2019 Jul 30;10:950. doi: 10.3389/fpls.2019.00950 (PMC6682694; doi:10.3389/fpls.2019.00950)
Supplement: Supplementary file 2 [file Data_Sheet_1.PDF]

Supplemental Data

I. Additional information about GeiEtr1

Sequence ID and link to information at NCBI:

gi|516257932|WP_017661895.1

Link to information on the JGI website:

https://img.jgi.doe.gov/cgi-bin/m/main.cgi?section=GeneDetail&page=geneDetail&gene_oid=2510103509

DNA sequence of *GeiEtr1*

ATGTGGGAAACTGTCAAAACTTTATTTTCACCGACGCAGTATATGCCGCA
CGGTCATTGTTACCTGTGGCAAACTCCGCTGGTTTGGTTGCACGTCGTTA
GCGACTCGCTCATTGCCGTGGCCTACTTGTCCATTCCGGCGATGCTGCTG
TACTTCGTGTTTCGGCGGCAGGACATACCGTTTTTGAATGTTTTCGTCAT
GTTCGGTGCGTTCATCGTGTTGTGCGGCGTCGGCCATTTATTCGACGTTT
GGACGTTGTGGCATCCTGCCTATTGGCTGTCGGGGGTCGAACGGGCTGCT
ACGGCTCTCATTTCCTGTTACACTGCCGGATCGATGGTGACGCTGCTGCC
TCGGTTTCTCTCCCTAAGAACTCCCGAAGAACTTGAAGCGATTAACCGAA
AACTCGAGCGGGAAGTCGATCGCCGCCGTCAAACTGAAGAAGCCTTACGA
AACATCGTCAAGGGAACAGCATCGGTGACGGGAGAGGAATTTTTTCCCGC
ATTAGTCCGCCATCTTTCCCAAGCGATCGACGTTCCCTATGCGTTGGTGT
CGGAAACTGTCGGCGATCCGCCTCACAAGTTACGGGTTCTAGCCAGTTGG
CAAAAAGGGCAGCTTTGCCAGGAAAAGGAGTACGATTTGGCAGGAACGCC
TTGCGAACAGGTGGTTTGCAGCGGTCGAGATTGCTATTATGCCGATAGCG
TCGCGGATCGATTTCCTCGCGATGCTGTACTCGATCGCATGAAAGCAACG
AGCTATTGGGGATTTCCCCTCTTCAACGTCTCGGGTGAAGTGGTGGGACA
TCTCTGCGCGATCGACACCCACCCTCCCCAAGATATCGATCGCACCCGCA
GCATTCTCGAAGTGTTTGCAGCTCGGGCGTCGGCGGAACTCGAACGTCAG
CACGTGACGGAAGCCCTGCAAGCGATGAATCGAGATCTCGAACGACGGGT
TGAGGAACGCACCGCCGAGGTCAAGCGCAGTAACGAAATGTTGGCAGGGA
AGGCGCAAGAATTGCAACAGGCGATCGATCGCCTGCAACGCACTCAAAGC
CAACTGATTCAATCGGAGAAAATGGCGTCGCTCGGCCAACTGGTGGCGGG
GGTGGCACACGAACTCAACAATCCCGTGAGCTTCGTTTACGGCAACATTA
CTTACGCCCAAGATTACAGCCGCGATCTGTTCGAGGTCTTGGAGATCTAT
CGACAACACTATCCCAACCCCACGCAGGCGGTGGCAGAGGCGATCGAACA
GGTCGATTTAGACTATATCCGACGAGACTTTCCCCGTTTGCTGGATTCGA
TGAAAACGGGTGCAAAACGCATTCAAAAGATCGTGGAGTCTCTTCGGAGC
TTTTCTCGACTCGACGAGGCTGAGTGCAAACGAGCCAACATCAACGCGGG
TATCGAGAGTACTTTAGAAATCGTGTCGGGACGGTTGAGCGCAACTCCAC
AACGACGAGCGATCGCCCTCGTCAAAGCGCTCGGTGACCTCCCTGAGATT
GAATGCTATCCCGGTCTGCTCAATCAAGCTGTGTTGAGCTTGCTCAATAA
CGCCATCGAAGTCTTGGAGGAACGGTTAAAACGCGAGCCAGATTTCACGC
CTCGTCTAGAAGTCACGACAGAAGTGATATCGAACGAAGCGTTTGGTAAA
GCGATCGCGATTGCCATCGGCGATAACGGCTTCGGCATCGACGAATCGAT
TCGAGATAAGATTTTCGATCCCTTTTTCACGACGAAACCCATTGGGAGAG
GCACGGGAATGGGATTGGCGAATGCCTATCAAATCGCGGTCGATCGCCAT
CGAGGACAGTTGCAGTGCATTTCGCACCCCGGTCGGGGAACGGTGTTTCA
GATCGAACTTCCCGTGTCTTACCACTGCGATGGAGCGAGTTCGGACGCCG
AGATGTTTCGCAGAGGCAAAGTACAAGGGAAATCGGAGAACGAATCCATC
CCGAAGCCTGCTGGGTAA

Predicted protein sequence for GeiEtr1

```
MWETVKTLFSPTQYMPHGHCYLWQTPLVWLHVVSDSLIAVAYLSIPAMLLYFVFRRQDIPFLNVFVMFGAFIV
LCGVGHLFDVWTLWHPAYWLSGVERAATALISCYTAGSMVTLLPRFLSLRTPEELEAINRKLEREVDRRRQTE
EALRNIVKGTASVTGEEFFPALVRHLSQAIDVPYALVSETVGDPPHKLRVLASWQKGQLCQEKEYDLAGTPCE
QVVCSGRDCYYADSVADRFPRDAVLDRMKATSYWGFPLFNVSGEVVGHLCAIDTHPPQDIDRTRSILEVFAAR
ASAELERQHVTEALQAMNRDLERRVEERTAEVKRSNEMLAGKAQELQQAIDRLQRTQSQLIQSEKMASLGQLV
AGVAHELNNPVSFVYGNITYAQDYSRDLFEVLEIYRQHYPNPTQAVAEAIEQVDLDYIRRDFPRLLDSMKTGA
KRIQKIVESLRSFSRLDEAECKRANINAGIESTLEIVSGRLSATPQRRAIALVKALGDLPEIECYPGLLNQAV
LSLLNNAIEVLEERLKREPDFTPRLEVTTEVISNEAFGKAIAIAIGDNGFGIDESIRDKIFDPFFTTKPIGRG
TGMGLANAYQIAVDRHRGQLQCISHPGRGTVFQIELPVSYHCDGASSDAEMFRRGKVQGKSENESIPKPAG
```

II. Additional information about GeiEtr2

Sequence ID and link to information at NCBI:

gi|5162574091|WP_017661454.1

Link to information on the JGI website:

https://img.jgi.doe.gov/cgi-bin/m/main.cgi?section=GeneDetail&page=geneDetail&gene_oid=2510103078

DNA sequence for *GeiEtr2*

```
ATGTGGACCGCTCTCGAATCGCTCCTGTCGCCAACTCAATATATGCCGCA
CGGCCAGTGTTACCTCTGGCAAACACCCCTCGTCTGGTTGCACGTCTTCA
GCGACAGTCTCATCGCCCTGTCTTACTATTCCATTCCCGGCCTTCTCGTC
TACTTCGTGCGTCAACGTCAAGACGTTCCCTTCCGCAGTGTATTTTGGCT
GTTCGGCGCGTTTATCGTCGCCTGCGGAACCGGCCATCTGCTCGAAGTCT
GGACGCTGTGGCATCCCGCCTACTGGCTCTCTGGCAGCGTCAAAGCCGTT
ACCGGATTAGTGTCTCTCTACACTGCCCTCACCCTCGTTCCCTTGCTTCC
CCAAGCCCTTGCATTGCCGAGTCCCGAACGACTGCGAACCATCAACCAAC
AACTCGAGTGCGAAATCCAGCACCGCAAAGAGGCTGAAGCCGCCCTGCGC
CAAGCCTACGATCGCCTCGAACAGCGAGTACGAGACGCCACCCAAGACTT
GCGCGATCGCACCACCGAACTGCTCGAGACCAACACCCACCTCGAAACAG
AAATCCTCCAGCGCCAAGACGCCGAAACTGCCCTGCGCCGGAGCGAAGAA
CGCTGGCAACTGGCCGTCCAAGGGAGCAACGAAGGCATTTGGGACTGGGA
CCTACAAAGCGATCGCGTGTTTTTCTCCGCCGGATGGAAAAAACTGCTCG
GCTACGAAGACGACGAAATCGCCGACGACCCGGGCGAATTTTGGACGCGC
CTCCATCCCGACGACATCGCCACCGTCCGCAACGCGATCGCCACCCATCT
CCAACAACAAACCGATTACACCGTCGAATTCCGAATGCAGTGCAAAGACA
GACGGTACAAATGGATACTCACGAGAGGCCGCGCCTGTTGGGACGACACC
GGACGCCCCATCCGTATGACTGGCTCCCACATCGACATCAGCGATCACAA
ACAGGCCGAGACGGCCTTACGAGAAAGCGCCGCTCGCGAACGAGCCATCG
CCCGCATCATCACGCGCATGAGAAAAACGCTCGACCTCGAGAAGATCTTC
AGCGCCACCACCGAAGAACTCCGAAACGCTCTCGAGTGCGATCGCGTTCT
CGTCTACCGCTTTCTCCCCGACTGGAGTGGAACCTACGTCGCCGAATCCG
TCATCCAAACGTACCACCCCCTCATCGATACCGCCAACCTACATCCGAAA
CTGACTCGCGTTGCCGTCCAGCGCGTCGGCTGTGCGGCCAGTATCCTCGA
AAGTGCCGACGATATCGTCCAAGACACCTATCTTCAAGAAACCCAAGGTA
GCTTCTACCGCCAAGGCAAAACCTATCGCGCCGTCACCGACATCTACCAC
GCTGGATTCGACGATTGCTATCTCGAACTCCTCGAACAGCTCGAGGCTCG
GGCTTATATTATCGTGCCGATTTTTTGCGGAAAAACCCTCTGGGGACTGC
TGGCCACCTACCAAAACGACAGACCGCGCCAGTGGACGGCCGCCAACATC
AAAATCGTCGTGCAAATCGGCTCGCAACTCGGCGTCGCCGTACAACAAGC
CGAACTGCTGGCGCGAACCCAGCAGCAAGCCGTCGAACTCCAGCAAGCTA
AGGAAACGGCCGAAGCCGCAAACCGGGCCAAAAGCGAATTTCTGGCCAAC
ATGAGCCACGAACTGCGAACGCCCCTCAACGCGATTCTCGGCTTTACACA
ACTGTTGCAGCGACAGTTTTCGAGTGCTTCCGAGCAGGGACAACATCTCG
AAACGATCGCCGGGAGCGGCGAACACCTGCTCGACTTGGTCAACGAAATT
TTAGATATGTCTAAAATCGAAGCCGGACGCGTCGAACTCGAAGAACGTAA
TTTCGACCTGTACCGATTCTGCGATCGCTTAGCCAGTATGCTACACCTGA
AAGCACAGAGTAAAAGACTGAGGCTTCACTTCGACATTGAAGAAACCGTT
CCTCAATTTATCCGAGCCGACGATCGCAAACTCCAACAAGTGGCCGTCAA
CCTGTTAGGAAACGCCCTCAAATTCACCGAAACCGGGAGCGTCACCTTCG
AGGTACGCCTGCGATCGCTCGATCCGATCGCCGCCTCCTGCGTGCTTCAC
TTTGCCGTTGAAGATACCGGCCCCGGCATCAGCGAGGACGAGCGAGCGTT
GTTGTTCGAGCCGTTCGTCCAAACTAGCACGGGTCTCCAGACCACCGGCG
GCACCGGTTTGGGACTGACCATCGGGCGGCGTTTCGTCCGATTAATGGGG
```

GGAGAAATGCAGGTCGAAAGCCAGGTCGGCCGGGGATCTCGGTTTTTCTT
CGACCTTCGAGTCAAACTGGCGATCGCCAGCGATCTCGTCTCTGAGGGAG
AGTTGCCCCACCGCTTGACCGTTCTCGCTCCAAACGAAACCATCTATCGG
ATTCTGGTCGCTGAAGACGAACCCACCAATCGCAGCTTGTTAGTCCGACT
GTTAAAAACCGTTGGCTTTGACGTGCGGGAAGCCGAAAACGGTCGAGCGG
CCGTTGACTGCTGGCGAGAATGGAAACCCCATCTCGTTTGGATGGATATG
CGAATGCCAGTTCTCGACGGTTACGAAGCCACCCGTACCATCAAAAACGC
CTCCAACGGGTGCGAGACGGTCGTTATCGCACTGACCGCCAGTGCCTTTG
AAGACGAACGACAGGCCGTCCTTGACGCCGGATGCGACGACTTCGTGCGA
AAACCCTATCGAGCGGCCGAAATTTTGGCGAAAATCCAGCAACATCTCGG
CGTCCAGTACGCCGACGACTCTGGCGATCTCAGCGACGATCTCGTAAAGG
AATCGTTGTCTGAAACCGACGAGCGGGACTCGATCGAATTCCAAGGAGAT
CGGACAATAGAGTGCGATCGCATCCCTGCCGAATGGCTCGAGCGTTTGCA
CGACGCCGCTCTCCAAGGGAGTGACGATAGCGTTTTCGAGTTAGCGGCCG
AACTCCCCGAAACCGAAGCCTCTCTCGCGCGAACGTTAAGCGATTGGGCG
CGAAACTTTCGCTTCGATCGCATCCTGAACTTCATCGACCTGACGACACG
CGACCCGAACGAAATCCATGACTGCTGA

Predicted protein sequence for GeiEtr2

MWTALESLLSPTQYMPHGQCYLWQTPLVWLHVFSDSLIALSYYSIPGLLVYFVRQRQDVPFRSVFWLFGAFIV
ACGTGHLLEVWTLWHPAYWLSGSVKAVTGLVSLYTALTLVPLLPQALALPSPERLRTINQQLECEIQHRKEAE
AALRQAYDRLEQRVRDATQDLRDRTTELLETNTHLETEILQRQDAETALRRSEERWQLAVQGSNEGIWDWDLQ
SDRVFFSAGWKKLLGYEDDEIADDPGEFWTRLHPDDIATVRNAIATHLQQQTDYTVEFRMQCKDRRYKWILTR
GRACWDDTGRPIRMTGSHIDISDHKQAETALRESAARERAIARIITRMRKTLDLEKIFSATTEELRNALECDR
VLVYRFLPDWSGTYVAESVIQTYHPLIDTANLHPKLTRVAVQRVGCAASILESADDIVQDTYLQETQGSFYRQ
GKTYRAVTDIYHAGFDDCYLELLEQLEARAYIIVPIFCGKTLWGLLATYQNDRPRQWTAANIKIVVQIGSQLG
VAVQQAELLARTQQQAVELQQAKETAEAANRAKSEFLANMSHELRTPLNAILGFTQLLQRQFSSASEQGQHLE
TIAGSGEHLLDLVNEILDMSKIEAGRVELEERNFDLYRFCDRLASMLHLKAQSKRLRLHFDIEETVPQFIRAD
DRKLQQVAVNLLGNALKFTETGSVTFEVRLRSLDPIAASCVLHFAVEDTGPGISEDERALLFEPFVQTSTGLQ
TTGGTGLGLTIGRRFVRLMGGEMQVESQVGRGSRFFFDLRVKLAIASDLVSEGELPHRLTVLAPNETIYRILV
AEDEPTNRSLLVRLLKTVGFDVREAENGRAAVDCWREWKPHLVWMDMRMPVLDGYEATRTIKNASNGCETVVI
ALTASAFEDERQAVLDAGCDDFVRKPYRAAEILAKIQQHLGVQYADDSGDLSDDLVKESLSETDERDSIEFQG
DRTIECDRIPAEWLERLHDAALQGSDDSVFELAAELPETEASLARTLSDWARNFRFDRILNFIDLTTRDPNEI
HDC
